# Quality of care for patients with type 2 diabetes in general practice according to patients' ethnic background: a cross-sectional study from Oslo, Norway

**DOI:** 10.1186/1472-6963-10-145

**Published:** 2010-05-28

**Authors:** Anh T Tran, Lien M Diep, John G Cooper, Tor Claudi, Jørund Straand, Kåre Birkeland, Wibeche Ingskog, Anne K Jenum

**Affiliations:** 1 Section of General Practice, Institute of Health and Community, University of Oslo, Oslo, Norway; 2 The Research Department, Oslo University Hospital, Aker, Oslo, Norway; 3 Department of Medicine, Stavanger University Hospital, Stavanger, Norway; 4 Department of Medicine, Nordland Hospital, Bodø, Norway; 5 Oslo Diabetes Research Centre, Oslo University Hospital, Aker, Oslo, Norway

## Abstract

**Background:**

In recent decades immigration to Norway from Asia, Africa and Eastern Europe has increased rapidly. The aim of this study was to assess the quality of care for type 2 diabetes mellitus (T2DM) patients from these ethnic minority groups compared with the care received by Norwegians.

**Methods:**

In 2006, electronic medical record data were screened at 11 practices (49 GPs; 58857 patients). 1653 T2DM patients cared for in general practice were identified. Ethnicity was defined as self-reported country of birth. Chi-squared tests, one-way ANOVAs, multiple regression, linear mixed effect models and generalized linear mixed models were used.

**Results:**

Diabetes was diagnosed at a younger age in patients from the ethnic minority groups (South Asians (SA): mean age 44.9 years, Middle East/North Africa (MENA): 47.2 years, East Asians (EA): 52.0 years, others: 49.0 years) compared with Norwegians (59.7 years, p < 0.001). HbA1c, systolic blood pressure (SBP) and s-cholesterol were measured in >85% of patients in all groups with minor differences between minority groups and Norwegians. A greater proportion of the minority groups were prescribed hypoglycaemic medications compared with Norwegians (≥79% vs. 72%, p < 0.001). After adjusting for age, gender, diabetes duration, practice and physician unit, HbA1c (geometric mean) for Norwegians was 6.9% compared to 7.3-7.5% in the minority groups (p < 0.05). The proportion with poor glycaemic control (HbA1c > 9%) was higher in minority groups (SA: 19.6%, MENA: 18.9% vs. Norwegians: 5.6%, p < 0.001. No significant ethnic differences were found in the proportions reaching the combined target: HbA1c ≤ 7.5%, SBP ≤ 140 mmHg, diastolic blood pressure (DBP) ≤ 85 mmHg and total s-cholesterol ≤5.0 mmol/L (Norwegians: 25.5%, SA: 24.9%, MENA: 26.9%, EA: 26.1%, others:17.5%).

**Conclusions:**

Mean age at the time of diagnosis of T2DM was 8-15 years younger in minority groups compared with Norwegians. Recording of important processes of care measures is high in all groups. Only one in four of most patient groups achieved all four treatment targets and prescribing habits may be sub-optimal. Patients from minority groups have worse glycaemic control than Norwegians which implies that it might be necessary to improve the guidelines to meet the needs of specific ethnic groups.

## Background

In Europe ethnic differences in the prevalence of diabetes have been described [1,2]. Ethnicity may be defined as the social group a person belongs to because of a shared culture, history, geographical origins, language, diet, physical, genetic and other factors [3].

In the developing world, both urbanisation and a shift towards a westernised lifestyle are risk factors for developing type 2 diabetes mellitus (T2DM). Immigrants from Asia and Africa living in Europe also have different diabetes characteristics than Europeans [4]. Inherited insulin resistance, low birth weight, migration itself as well as lifestyle, cultural and socioeconomic factors may contribute to the observed differences [5-7].

In T2DM, a long-term, intensified intervention targeting multiple risk factors is crucial for reducing cardiovascular death rates and micro-vascular complications [8,9]. Across Europe, the structure of care for T2DM varies by country, from mainly hospital based to mainly general practitioner (GP) based [10].

During the last three decades, immigration to Norway from Asia, Africa, Eastern Europe and Central- and South America has increased rapidly. In 2005 ethnic groups with origin from these regions (first and second generation) accounted for approximately 18% of the population in Oslo [11].

Socioeconomic status, chronic illnesses and risk factors for diabetes (e.g., obesity and physical inactivity), and the use of health care services differ between and within these groups [12]. In 2000, an alarmingly high prevalence of diabetes was found among 30-59 year old South Asians compared with Norwegians, 14.3% vs. 5.9% in men and 27.5% vs. 2.9% in women [13].

In Norway, the majority of T2DM patients are cared for in general practice whereas type 1 diabetes mellitus (T1DM) patients usually receive hospital based specialist care. In general, the quality of care for T2DM has improved since 1995 [14,15]. However, little is known about the ability of GPs to adapt their clinical practice to provide optimal health care for all diabetic patients irrespective of their ethnic origin.

The aim of this study was to assess the quality of care for different ethnic minority patient groups with T2DM treated in general practice in Oslo and to compare it with the quality of care received by Norwegians.

## Methods

### Design, participants and setting

This study is based on the Oslo data subset from a national cross-sectional study assessing the quality of diabetes care in general practice [14]. In 2006 retrospective data were captured from electronic medical records (EMR) in practices in four areas in Norway. 17 practices located in a suburb in eastern Oslo where 31% of the population are ethnic minorities [11] were invited to participate in this study. 14 practices accepted the invitation and 11 took part in the study. Of the three practices accepting but not participating, one could not participate due to problems with the EMR system and two small practices were not included due to shortage of funding. The data used in this study are therefore based on 11 practices (49 GPs, 58857 patients).

A software program designed for the study (Mediata AS, Norway) was used to identify patients with diabetes and to capture predefined data from the GPs' EMR (from 2003 to 2005).

In total, 2064 patients with a diabetes diagnosis were identified. For each identified patient, data was manually checked by an experienced research nurse (WI) to identify patients who met our inclusion criteria. Because the study addressed quality of care in general practice for T2DM patients, we excluded patients with T1DM, patients with T2DM who had two or more visits in hospital clinics for diabetes care in the previous 12 months, those who had less than six months of follow-up, those who had moved, were deceased, or had missing data regarding country of birth.

The study was approved by the Regional ethics board for biomedical research, the Directorate for Health and Social Affairs and the Data Inspectorate.

### Variables

Ethnicity was based on self-reported country of birth, as noted in the EMR. Included patients were categorized as follows: Norwegians (including ≈2% Scandinavians and others from Western Europe/North America), South Asians (SA; ≈75% Pakistanis, ≈25% Sri Lankans and a few Indians), Middle East/North Africans (MENA; mainly from Turkey and Morocco), East Asians (EA; mainly from Vietnam), and others (O; from Eastern Europe, South Sahara Africa, Central- and South America), with all except those categorized as Norwegians are referred to as ethnic minorities.

Demographic characteristics and data on processes of care, intermediate outcomes of care and prescriptions for oral hypoglycaemic agents (OHAs), insulin, anti-hypertensive- or statin therapy were recorded. Processes of care data include whether or not a recommended measurement/examination such as HbA1c, systolic blood pressure (SBP), diastolic blood pressure (DBP), s-cholesterol, microalbuminuria, referral to ophthalmologist, foot-examination, smoking habits, body height and body weight had been performed in the defined period. Intermediate outcomes were recorded results of particular measurements or examinations. Data regarding HbA1c, SBP, DBP, microalbuminuria, body weight and foot-examinations were from 2005, data for eye examinations from 2004 or 2005, and for s-cholesterol and smoking habits from the years 2003 to 2005. National guidelines recommend that these measures should be performed on a regular basis [16]. The most recent result was chosen if more than one value was available.

The treatment targets used in this study were: HbA1c ≤ 7.5%, SBP ≤ 140 mmHg, DBP ≤ 85 mmHg, and total s-cholesterol ≤ 5.0 mmol/L [14,15].

### Statistical analyses

Patient's age when the diabetes diagnosis was established, was estimated based on data regarding disease duration.

Chi-squared tests were applied for testing differences between proportions in the ethnic groups. Differences in mean values for the continuous variables between the ethnic groups were tested by one-way ANOVAs. Multiple regression models were applied to estimate ratios of geometric means and mean differences for the continuous variables in the particular group compared with the reference group (Norwegians). Random intercept models were used for adjustment for physicians and practices. Physicians and practices were specified as random and fixed effect variables correspondingly in linear mixed effect models (lme) and generalized linear mixed models (glmmML). The lme and glmmML functions were used for continuous and binary dependent variables respectively.

Because the data were highly skewed, HbA1c values were log-transformed before applying the random intercept model, and the results (estimates with 95% confidence intervals) were transformed back to the original scale using anti-log. HbA1c (geometric mean) was used for comparison between groups.

Two-sided tests were used, and p-values were not corrected for multiple testing.

The analyses were performed with R 2.8.1 [17] and SPSS 15.0 for Windows. The lme and glmmML functions were from the nlme and glmmML packages in R.

## Results

We identified 2064 patients with diabetes from 58857 EMR. A total of 411 patients were excluded (264 had two or more visits in hospital based care the previous year, 128 had moved, were deceased or for other reasons had less than 6 months follow-up in the practice, 17 had T1DM and there were missing data on country oft birth in two patients).

Of the 1653 included T2DM patients, 68.5% were classified as Norwegians and 31.5% were ethnic minorities (Table 1). The groups differed significantly for mean age, age at diagnosis, and body mass index (BMI) (Table 1).

**Table 1:** Characteristics of patients (n = 1653) with type 2 diabetes mellitus by ethnic background.

***Characteristics***	***All (n = 1653)***	***Norwegians (n = 1129)***	***South Asians (n = 322)***	***MENA***^**a **^***(n = 81)***	***East Asians (n = 54)***	***Others***^**b **^***(n = 67)***	**P**^**c**^
**Males ***(n=)*	828	564	160	42	21	41	0.19

**Age**							
Valid cases *(n=)*	1653	1129	322	81	54	67	
Mean (year)		66.4	52.3	53.1	57.7	56.6	<0.001
95% CI		65.7 to 67.2	51.2 to 53.3	50.9 to 55.3	54.9 to 60.6	53.6 to 59.6	

**Age at diagnosis**							
Valid cases *(n=)*	1526	1061	292	71	44	58	
Mean (year)		59.7	44.9	47.2	52.0	49.0	<0.001
95% CI		58.9 to 60.5	43.8 to 46.0	44.8 to 49.6	48.4 to 55.5	45.9 to 52.1	

**Diabetes duration**							
Valid cases *(n=)*	1526	1061	292	71	44	58	
Mean (year)		6.6	7.0	5.5	5.0	6.4	0.11
95% CI		6.2 to 6.9	6.3 to 7.7	4.4 to 6.7	3.8 to 6.1	5.0 to 7.8	

**Body Mass Index**							
Valid cases *(n=)*	723	501	140	37	23	22	
Mean (kg/m2)		30.1	29.8	31.9	27.5	29.1	<0.001
95% CI		29.6 to 30.6	29.0 to 30.7	29.4 to 34.4	26.0 to 28.9	26.6 to 31.6	

^a ^Patients from Middle East/North Africa.

^b ^Patients from other regions.

^c ^p-values. One-way ANOVAs were applied to compare mean age, diabetes duration, age at diagnosis and BMI between ethnic groups. Chi-square test was applied to compare percentage of males between ethnic groups.

Among patients with body height and weight recorded, 46% of Norwegians, 43% of SA, 60% of MENA, 17% of EA and 41% of patients from other regions had BMI ≥ 30 kg/m^2^. When applying the definition of obesity in Asians as suggested by WHO, 81.4% of SA and 73.9% of EA had BMI ≥ 25 kg/m^2 ^[18].

### Processes of care

In all groups, 85-97% had their HbA1c, SBP and s-cholesterol measured, 34-50% had their microalbuminuria assessed, and 50-63% had been referred to an ophthalmologist. Foot assessment was recorded in less than 20% (Table 2). Blood pressure (BP) and smoking habits were recorded significantly less often whereas s-cholesterol level was measured more often in the ethnic minorities compared with Norwegians.

**Table 2:** Proportions (%) of patients with important processes of care recorded in electronic medical records.

***Features recorded in EMR***	***All (n = 1653)***	***Norwegians (n = 1129)***	***South Asians (n = 322)***	***MENA***^**a **^***(n = 81)***	***East Asians (n = 54)***	***Others***^**b **^***(n = 67)***	**p**^**c**^
HbA1c	94.7	95.2	93.5	91.4	96.3	94.0	0.45

Systolic blood pressure	90.6	92.5	85.7	87.7	85.2	91.0	0.003

S-Cholesterol	94.2	92.3	96.9	95.1	94.4	97.0	0.03

Micro-albuminuria	46.2	46.8	46.9	43.2	50.0	34.3	0.33

Reference to ophthalmologist	60.3	60.4	63.4	49.5	59.3	58.2	0.25

Foot examination	18.0	18.7	18.0	14.8	11.1	14.9	0.54

Smoking habits	59.4	64.0	53.4	42.0	46.3	43.3	<0.001

Body height	47.1	47.5	46.3	53.1	48.1	35.8	0.31

Body weight	56.1	56.8	57.8	53.1	50.0	46.3	0.37

^a ^Patients from Middle East/North Africa.

^b ^Patients from other regions.

^c ^p-values. Chi-squared tests were applied to identify differences between ethnic groups with recorded measurement.

### Medication use and intermediate outcomes

OHAs alone or in combination with insulin were prescribed more often to ethnic minorities (SA: 84%, MENA: 79%, EA: 80%, other regions: 81%) than in Norwegians (72%) (Table 3). Compared with SA, almost twice as many Norwegians were not prescribed any hypoglycaemic agent (p < 0.001).

**Table 3:** Proportions (%) of patients receiving hypoglycaemic, anti-hypertensive and statin therapy.

***Treatment groups***	***All (n = 1653)***	***Norwegians (n = 1129)***	***South Asians (n = 322)***	***MENA***^**a **^***(n = 81)***	***East Asians (n = 54)***	***Others***^**b **^***(n = 67)***	**P**^**c**^
Diet alone	24.7	27.9	16.1	21.0	20.4	19.4	<0.001

Oral agents	56.1	54.8	57.8	61.7	61.1	58.2	0.19

Insulin and oral agents	9.5	7.5	16.1	7.4	5.6	16.4	0.001

Insulin alone	9.7	9.7	9.9	9.9	13.0	6.0	0.66

Anti-hypertensive therapy	61.1	69.4	43.2	34.6	44.4	53.7	<0.001

Statin therapy	39.0	42.8	35.7	24.7	22.2	22.4	<0.001

^a ^Patients from Middle East/North Africa.

^b ^Patients from other regions.

^c ^p-values. Chi-squared tests were applied to identify differences between ethnic groups receiving treatment.

HbA1c (geometric mean) was 6.9% in Norwegians and 7.1 to 7.6% in the minority groups. After adjustments for confounders, i.e. age, gender, diabetes duration and clustering variables, i.e. practice unit and physician unit all the ethnic minority groups had significantly higher mean levels of HbA1c (7.3-7.5%) compared to Norwegians (6.9%), p < 0.05 (Table 4). Diabetes duration and practice were significant predictors for HbA1c.

**Table 4:** Crude and adjusted means with 95% CIs for risk factors in five patient groups.

***Variable***^**a**^	***Ethnic groups ***^**b**^	***Unadjusted***^**d**^			***Adjusted ***^**g**^			***Adjusted***^**h**^		
		
		**Mean**^**c**^	**Ratio/Difference**^**e **^**(95% CI)**	**P **^**f**^	**Mean**^**c**^	**Ratio/Difference**^**e **^**(95% CI)**	**P **^**f**^	**Mean**^**c**^	**Ratio/Difference**^**e **^**(95% CI)**	**P **^**f**^
HbA1c (%)										
	Nor	6.91	Ref.		6.89	Ref		6.94	Ref	
	SA	7.55	1.09 (1.07 to 1.12)	<0.001	7.47	1.08 (1.06 to 1.11)	<0.001	7.46	1.07 (1.05 to 1.10)	<0.001
	MENA	7.08	1.03 (0.99 to 1.07)	0.195	7.14	1.04 (1.00 to 1.10)	0.086	7.32	1.05 (1.01 to 1.10)	0.008
	EA	7.15	1.04 (0.99 to 1.08)	0.120	7.28	1.06 (1.01 to 1.11)	0.026	7.38	1.06 (1.01 to 1.11)	0.011
	Others	7.33	1.06 (1.02 to 1.11)	0.004	7.31	1.06 (1.02 to 1.11)	0.007	7.33	1.06 (1.01 to 1.10)	0.011

SBP (mmHg)										
	Nor	138.9	Ref		137.5	Ref		137.8	Ref	
	SA	126.7	-12.2 (-14.5 to -10.0)	<0.001	130.7	-6.8 (-9.4 to -4.3)	<0.001	131.0	-6.8 (-9.4 to -4.2)	<0.001
	MENA	128.4	-10.5 (-14.6 to - 6.3)	<0.001	132.2	-5.3 (-9.6 to -1.0)	0.015	132.8	-5.0 (-9.3 to -0.6)	0.024
	EA	132.7	-6.2 (- 11.3 to -1.2)	0.016	137.0	-0.6 (-6.1 to 5.0)	0.842	137.9	0.1 (-5.5 to 5.6)	0.981
	Others	131.1	-7.8 (-12.2 to -3.4)	0.001	134.4	-3.2 (-7.8 to 1.5)	0.181	135.5	-2.3 (-6.9 to 2.4)	0.340

DBP (mmHg)										
	Nor	79.6	Ref		80.2	Ref		80.2	Ref	
	SA	76.7	-2.9 (-4.1 to -1.6)	<0.001	75.8	-4.3 (-5.7 to -2.9)	<0.001	76.2	-4.0 (-5.4 to -2.5)	<0.001
	MENA	76.6	-3.0 (-5.2 to -0.8)	0.008	75.3	-4.8 (-7.2 to -2.5)	<0.001	75.8	-4.4 (-6.7 to -2.0)	<0.001
	EA	80.4	0.8 (-2.0 to 3.5)	0.579	80.8	0.6 (-2.5 to 3.6)	0.709	81.4	1.2 (-1.8 to 4.2)	0.430
	Others	76.0	-3.6 (-5.9 to -1.2)	0.004	75.5	-4.6 (-7.2 to -2.1)	<0.001	76.5	-3.7 (-6.3 to -1.2)	0.004

S-Chol (mmol/L)										
	Nor	5.15	Ref		5.11	Ref		5.14	Ref	
	SA	4.92	-0.22 (-0.36 to -0.09)	0.001	5.00	-0.12 (-0.27 to 0.04)	0.139	5.06	-0.08 (-0.24 to 0.08)	0.313
	MENA	5.15	0.01 (-0.24 to 0.25)	0.962	5.14	0.03 (-0.23 to 0.29)	0.823	5.23	0.09 (-0.18 to 0.35)	0.526
	EA	5.40	0.25 (-0.05 to 0.55)	0.099	5.45	0.33 (0.01 to 0.66)	0.046	5.51	0.37 (0.04 to 0.70)	0.026
	Others	5.14	-0.01 (-0.27 to 0.26)	0.652	5.16	0.05 (-0.23 to 0.33)	0.725	5.20	0.06 (-0.23 to 0.34)	0.702

^a ^HbA1c was log-transformed before applying the random intercept model for estimation due to highly skewed distribution of data. The results (estimates with 95% confidence intervals) were transformed back to the original scale using anti-log. SBP: systolic blood pressure; DBP: diastolic blood pressure; S-Chol: S-Cholesterol.

^b ^Nor: Norwegians; SA: South Asians, MENA: patients from Middle East/North Africa, EA: East Asians, Others: patients from other regions.

^c ^Geometric means of HbA1c and arithmetic means of SBP, DBP and S-Cholesterol.

^d ^Multiple regression models were applied to estimate difference in the particular group compared to the reference group (Norwegians).

^e ^The antilog of the effect of the ethnic group on HbA1c is the ratio of geometric means between the particular group and the reference group (Norwegians).

^f ^p-values.

^g ^Multiple regression models were applied to estimate difference in the particular group compared to reference group (Norwegians) adjusted for confounders (age, gender, diabetes duration).

^h ^Linear mixed effect models (lme in R [17]) were applied to estimate difference in the particular group compared to reference group (Norwegians) adjusted for confounders (age, gender, diabetes duration), practices and physicians (as random intercept).

Fewer in the ethnic minority groups were prescribed anti-hypertensive agents or statins (p < 0.001) (Table 3). SA had lower SBP and DBP values than Norwegians (Table 4). The differences remained significant after adjustments for confounders as well as after additional adjustment for clustering. Only EA had significantly higher s-cholesterol compared with Norwegians after adjustments for confounders and clustering.

### Achievement of national targets

**Figure 1:**
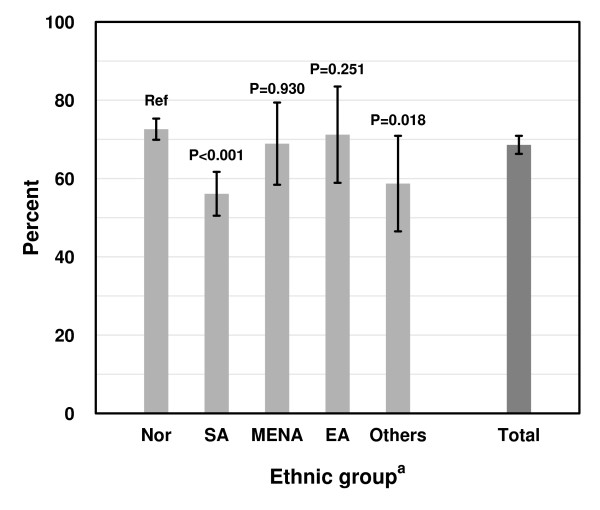
**Achievement (% with 95% CI) of national target for HbA1c (≤7.5%) by ethnicity**. ^a ^Nor = Norwegians; SA = South Asians; MENA = Middle East/North Africa; EA = East Asia; Others = patients from other regions.

While 72.6% of Norwegians had HbA1c ≤ 7.5%, the corresponding proportion varied from 56.1% to 71.2% in the minority groups (p < 0.001 for between group differences) (Figure 1 and Table 5).

**Table 5:** Proportions (%) reaching national treatment goals in five patient groups.

*Treatment targets*	*All (n = 1653)*	*Norwegians (n = 1129)*	*South Asians (n = 322)*	***MENA***^**b **^***(n = 81)***	*East Asians (n = 54)*	***Others***^**c **^***(n = 67)***	**P**^**d**^
HbA1c (≤7.5%)	68.6	72.6	56.1	68.9	71.2	58.7	<0.001

SBP^a ^(≤140 mmHg)	69.6	63.6	86.2	80.3	71.7	82.0	<0.001

DBP^a ^(≤85 mmHg)	80.6	79.0	84.7	87.3	73.9	86.9	0.048

S-Cholesterol (≤5.0 mmol/L)	49.7	49.3	55.1	44.2	35.3	47.7	0.061

All four targets	25.1	25.5	24.9	26.9	26.1	17.5	0.77

^a ^SBP: systolic blood pressure; DBP: diastolic blood pressure.

^b ^Patients from Middle East/North Africa.

^c ^Patients from other regions.

^d ^p-values. Chi-squared tests were applied to identify differences between ethnic groups achieving the national targets.

Poor glycaemic control (HbA1c > 9.0%) was three times more prevalent among patients from SA (19.6%), from MENA (18.9%) and from other regions (17.5%) than in Norwegians (5.9%), (p < 0.001). An exception was patients from EA, where only 3.8% had poor control. When including the treatment targets for SBP, DBP and serum cholesterol, 17.5 to 26.9% of all groups had reached all four targets (Table 5).

Compared with Norwegian patients odds ratio (OR) for achieving good glycaemic control (HbA1c ≤ 7.5%) adjusted for confounders and clustering was reduced in all ethnic minority groups (SA: 0.6 (0.5 to 0.9), MENA: 0.6 (0.4 to 1.1) EA: 0.7 (0.3 to 1.4), O: 0.6 (0.3 to 1.1)). Compared with the Norwegian group only the O group had a significantly reduced OR for achieving the combined (all-four) target adjusted for the same factors (SA: 0.8 (0.5 to 1.1), MENA: 0.7 (0.4 to 1.3) EA 0.6 (0.3 to 1.4), O 0.4 (0.2 to 0.9).

## Discussion

This is the first Norwegian study assessing quality of care parameters in patients with T2DM treated in general practice according to their ethnic background. In general, the GPs performed the recommended measures at comparable rates in both Norwegians and ethnic minority patients. However, in all minority groups the glycaemic control was poorer than in Norwegians (adjusted geometric mean HbA1c 0.4-0.6% higher) despite more intensive treatment with OHAs and/or insulin. A younger age at time of diagnosis (8-15 years) was found in the ethnic minority groups.

Ethnic minority groups accounted for 32% of the patients with T2DM, a figure that is very similar to the percentage of ethnic minorities in the background population in the area (31%) [11]. We found a prevalence of T2DM for the population >20 years of age of 4.6% and for the total study population of 3.5%. This corresponds well with earlier studies from the same area with 4.1% (age group 30-67 years of age) reporting known diabetes in 2000 [13]. The prevalence may appear to be lower than expected in a multiethnic population, but the young age of the ethnic minority population must be taken into account. We thus consider the study population to be fairly representative of the suburban area in eastern Oslo. The list based practice system allows us to trace patients and ensures that there are no duplicates. Manual verification of the electronically extracted data by one experienced nurse contributes to the internal validity of this study.

The younger age at diagnosis in the minority groups compared with Norwegians, indicates that the pathophysiological processes of T2DM start or accelerate earlier. The difference in age at diagnosis between SA and the majority population was even larger in our study (15 years) than in two studies from the UK (11 and 5 years respectively) [19,20].

The quality of care in terms of processes of care was comparable to that found in other Norwegian regions and in the UK [14,21]. HbA1c, BP and s-cholesterol were measured in the majority of patients in nearly all groups as recommended by the national guidelines. These results may reflect a relatively well-developed health care system, implying equal access to the public health service for all and a personal GP for everyone according to a list based system.

However, disparities in intermediate outcomes such as HbA1c appear to be more difficult to eliminate. Differences in metabolic and lifestyle factors like degree of insulin resistance, physical inactivity and diet together with compliance with prescribed antihyperglycaemic medication, may contribute [22,23]. In addition to ethnicity and diabetes duration the practice unit was a significant parameter in the final model of the multiple regression analyses and organisational matters like the staffing of practices and lack of effective cooperation between GPs and secondary care may contribute to poorer diabetes control in the ethnic minority groups. Language barriers and cultural factors affecting disease perceptions and the ability to cope with the self-management strategy of diabetes care, may also have contributed to the observed differences in the intermediate outcomes [24].

The ethnic differences in glycaemic control, especially between SA and Norwegians were comparable to corresponding reports from the UK [19,20,25]. Although SA have lower mean SBP and DBP compared to Norwegians, the GPs should pay particular attention to this ethnic group because of their higher risk for coronary heart disease [5,20].

In our study, only one out of four in most groups reached the combined goals for glycaemia, SBP, DBP and lipid control although a larger proportion of the minority groups achieved the treatment target for SBP [26].

After implementation of pay-for-performance in the UK, the proportion of diabetic patients reaching treatment target for HbA1c, BP and total cholesterol increased across the ethnic groups. However, poorer glycaemic control in South Asian and black African and black Caribbean groups persisted relative to the white British group [27].

In Norway, the high level of performed measurements for HbA1c, SBP and s-cholesterol and the similar intermediate outcomes were achieved without specific financial incentives. In 2008, a small additional payment for using an annual structured electronic diabetes control form was introduced [28] which may stimulate the GPs to follow the guidelines and thereby improve the quality of diabetes care in the long term. Nevertheless it is essential to monitor the ethnic disparities in intermediate outcomes for diabetes over time [29].

## Limitations

The study has limitations due to the small sample size for some of the groups and the heterogeneity within groups.

Furthermore we lack potential important information on socioeconomic status at the individual level. We do not have data to explore the reasons for poor glycaemic control in the minority groups, such as compliance to prescribed medication and the impact of language barriers.

Our data do not allow us to estimate the T2DM prevalence in the different ethnic groups.

The cross-sectional design implies that we have not studied in the influence of ethnicity on process of care or intermediate outcome over time.

## Conclusion

Our findings show that GPs in eastern Oslo deliver comparable quality of diabetes care to all ethnic patient groups with respect to process measures.

However, this study has identified important challenges in the treatment of hyperglycaemia in the minority groups. The early onset of T2DM and the poor glycaemic control of relatively young ethnic minority patients represent a major concern. It underlines the need for early diagnosis, a tight follow-up of ethnic minority T2DM patients and a tailored collaboration between GPs and diabetic specialists for patients who do not achieve treatment goals [30,31].

## Competing interests

The authors declare that they have no competing interests.

## Authors' contributions

The study was conceived and designed by AKJ, TC, JGC. WI collected the data. ATT and LMD performed the analysis. All authors participated in discussing the results. ATT wrote the first draft of the manuscript and all authors commented on the drafts and approved the final version.

## Authors' Information

Anh Thi Tran, MD is a qualified specialist family medicine (GP) and a research fellow at Section of General Practice, Institute of Health and Community, University of Oslo. Lien My Diep, MSc is a statistician at the research department, Oslo University Hospital, Aker, Oslo. John Grandham Cooper, MD is an endocrinologist at the Department of Medicine, Stavanger University Hospital, Stavanger. Tor Claudi, MD is an internist and a specialist in General practice, at the Department of Medicine, Nordland Hospital, Bodø. Jørund Straand, MD, PhD, is a professor in General Practice at the Section of General Practice, Institute of health and Community, University of Oslo. Kåre Birkeland, MD, PhD, is an Endocrinologist and Head of Department of Endocrinology at Oslo University Hospital, Aker and Professor of Endocrinology at Faculty of Medicine, University of Oslo. W. Ingskog was a research nurse at the Diabetes Research Centre, Oslo University Hospital, Aker, Oslo. Anne Karen Jenum, MD, PhD, is a Qualified Specialist Family Medicine (GP), Qualified specialist Public Health/Community Medicine, Supervisor Family Medicine and a post doctoral research fellow at Oslo Diabetes Research Centre, Oslo University Hospital, Aker, Oslo.

## Pre-publication history

The pre-publication history for this paper can be accessed here:

http://www.biomedcentral.com/1472-6963/10/145/prepub
